# Supplementary material for: A Systematic Review and Meta-Analysis on the Presence of Escherichia coli O157:H7 in Africa from a One Health Perspective
Source: Microorganisms. 2025 Apr 14;13(4):902. doi: 10.3390/microorganisms13040902 (PMC12029247; doi:10.3390/microorganisms13040902)
Supplement: Supplementary file 1 [file microorganisms-13-00902-s001.zip › Supplementary Table S2-S3.pdf]

A Systematic Review and Meta-Analysis on the presence of *Escherichia coli* O157:H7 in Africa from a One Health Perspective

Last Search Date: October 28, 2024

Table S2. Keywords and search terms used in the literature search

Electronic databases	Keywords and search terms	Records Found
PubMeb	OR OR OR OR OR OR OR OR OR OR OR OR OR) AND (Africa OR each African country name (((((((((((((((((*Escherichia coli* O157:H7) OR (*E. coli* O157:H7)) OR (One Health)) OR (clinical samples)) OR (human samples)) OR (animal samples)) OR (livestock samples)) AND (animal products)) OR (food products)) OR (water)) OR (wastewater)) OR (vegetables)) OR (farms)) OR OR (Multidrug-resistant)) OR (MDR)) OR (antibiotic resistance)) OR (antimicrobial resistance genes)) AND (Africa)) OR (country name))	543
Scopus	"*Escherichia coli* O157:H7" OR " *E. coli* O157:H7" OR " One Health " OR " clinical samples " OR " human samples" OR " animal samples " OR "livestock samples" OR "animal products" OR "food products" OR " water " OR " wastewater " OR " vegetables " OR " farms " OR "Multidrug-resistant" OR "MDR" OR "antibiotic resistance" OR "antimicrobial resistance genes" AND Africa" OR "country name"	598
Web of Science	"Escherichia coli O157:H7" OR " E. coli O157:H7" OR " One Health " OR " clinical samples " OR " human samples" OR " animal samples " OR "livestock samples" OR "animal products" OR "food products" OR " water " OR " wastewater " OR " vegetables " OR " farms " OR "Multidrug-resistant" OR "MDR" OR "antibiotic resistance" OR "antimicrobial resistance genes" AND Africa" OR "country name"	345
Google Scholar	*Escherichia coli* O157:H7, *E. coli* O157:H7, One Health, clinical samples, human samples, animal samples, livestock samples, animal products, food products, Environment samples, water, wastewater, vegetables, farms, Africa, each African country name	271
Total	**Total Records Found**	1757

Table S3: Joanna Briggs Institute critical appraisal checklist for studies reporting prevalence data

		Joanna Briggs Institute's critical appraisal questions												
**Author & Year**	**Total Sample Size**	**Q1**	**Q2**	**Q3**	**Q4**	**Q5**	**Q6**	**Q7**	**Q8**	**Q9**	**Score**	**% Yes**	**Stufy quality**	**Overall appraisal**
El-Gohary et al., 2020	200	**Y**	**Y**	**N**	**Y**	**N**	**Y**	**Y**	**Y**	**Y**	**9**	**77.8**	High	Included
Refaay et al., 2024	648	**Y**	**Y**	**Y**	**Y**	**Y**	**Y**	**Y**	**Y**	**Y**	**9**	**100**	High	Included
Selim et al., 2014	384	**Y**	**Y**	**Y**	**Y**	**Y**	**Y**	**Y**	**Y**	**Y**	**9**	**100**	High	Included
Mansour et al., 2023	550	**Y**	**Y**	**Y**	**Y**	**Y**	**Y**	**Y**	**Y**	**Y**	**9**	**100**	High	Included
Hassan Ali et al., 2010	103	**Y**	**Y**	**Y**	**Y**	**Y**	**Y**	**Y**	**Y**	**Y**	**9**	**100**	High	Included
Hassanien et al., 2020	300	**Y**	**Y**	**Y**	**Y**	**Y**	**Y**	**Y**	**Y**	**Y**	**9**	**100**	High	Included
Hamed et al., 2017	310	**Y**	**Y**	**Y**	**Y**	**Y**	**Y**	**Y**	**Y**	**Y**	**9**	**100**	High	Included
Ahmed et al., 2017	700	**Y**	**Y**	**Y**	**Y**	**Y**	**Y**	**Y**	**Y**	**Y**	**9**	**100**	High	Included
Mahmoud et al., 2020	230	**Y**	**Y**	**Y**	**Y**	**Y**	**Y**	**Y**	**Y**	**Y**	**9**	**100**	High	Included
Nehoya et al., 2020	270	**Y**	**Y**	**Y**	**Y**	**Y**	**Y**	**Y**	**Y**	**Y**	**9**	**100**	High	Included
Lupindu et al., 2014	1046	**Y**	**Y**	**Y**	**Y**	**Y**	**Y**	**Y**	**Y**	**Y**	**9**	**100**	High	Included
Mkala et al., 2017	307	**Y**	**Y**	**Y**	**Y**	**Y**	**Y**	**Y**	**Y**	**Y**	**9**	**100**	High	Included
Ajuwon et al., 2021	415	**Y**	**Y**	**Y**	**Y**	**Y**	**Y**	**Y**	**Y**	**Y**	**9**	**100**	High	Included
Akinduti et al., 2022	508	**Y**	**Y**	**Y**	**Y**	**Y**	**Y**	**Y**	**Y**	**Y**	**9**	**100**	High	Included
Ayodele et al., 2020	823	**Y**	**Y**	**Y**	**Y**	**Y**	**Y**	**Y**	**Y**	**Y**	**9**	**100**	High	Included
Ekundayo et al., 2012	366	**Y**	**Y**	**Y**	**Y**	**Y**	**Y**	**Y**	**Y**	**Y**	**9**	**100**	High	Included
Izevbuwa et al., 2020	50	**Y**	**Y**	**Y**	**Y**	**Y**	**Y**	**Y**	**Y**	**Y**	**9**	**100**	High	Included
Ujoh et al., 2022	349	**Y**	**Y**	**Y**	**Y**	**Y**	**Y**	**Y**	**Y**	**Y**	**9**	**100**	High	Included
Onwumere-Idolor et al., 2024	406	**Y**	**Y**	**Y**	**Y**	**Y**	**Y**	**Y**	**Y**	**Y**	**9**	**100**	High	Included
Chigor et al., 2010	340	**Y**	**Y**	**Y**	**Y**	**Y**	**Y**	**Y**	**Y**	**Y**	**9**	**100**	High	Included
Hammuel et al., 2014	160	**Y**	**Y**	**Y**	**Y**	**Y**	**Y**	**Y**	**Y**	**Y**	**9**	**100**	High	Included
Fuh et al., 2018	726	**Y**	**Y**	**Y**	**Y**	**Y**	**Y**	**Y**	**Y**	**Y**	**9**	**100**	High	Included
Abong'o et al., 2008c	900	**Y**	**Y**	**Y**	**Y**	**Y**	**Y**	**Y**	**Y**	**Y**	**9**	**100**	High	Included
Ateba et al., 2011	220	**Y**	**Y**	**Y**	**Y**	**Y**	**Y**	**Y**	**Y**	**Y**	**9**	**100**	High	Included

Ateba et al., 2013	220	**Y**	**Y**	**Y**	**Y**	**Y**	**Y**	**Y**	**Y**	**Y**	**9**	**100**	High	Included
Ateba et al., 2014	220	**Y**	**Y**	**Y**	**Y**	**Y**	**Y**	**Y**	**Y**	**Y**	**9**	**100**	High	Included
Bolukaoto et al., 2019	520	**Y**	**Y**	**Y**	**Y**	**Y**	**Y**	**Y**	**Y**	**Y**	**9**	**100**	High	Included
Duvenage et al., 2017	428	**Y**	**Y**	**Y**	**Y**	**Y**	**Y**	**Y**	**Y**	**Y**	**9**	**100**	High	Included
Msolo et al., 2016	252	**Y**	**Y**	**N**	**Y**	**N**	**Y**	**Y**	**Y**	**Y**	**7**	**77.8**	High	Included
Müller et al., 2018	403	**Y**	**Y**	**Y**	**Y**	**Y**	**Y**	**Y**	**Y**	**Y**	**9**	**100**	High	Included
Ogundare et al., 2024	537	**Y**	**Y**	**Y**	**Y**	**Y**	**Y**	**Y**	**Y**	**Y**	**9**	**100**	High	Included
Ateba et al., 2008	800	**Y**	**Y**	**N**	**Y**	**N**	**Y**	**Y**	**Y**	**Y**	**7**	**77.8**	High	Included
Momba et al., 2004	540	**Y**	**Y**	**Y**	**Y**	**Y**	**Y**	**Y**	**Y**	**Y**	**9**	**100**	High	Included
Myataza et al., 2017	288	**Y**	**Y**	**N**	**Y**	**N**	**Y**	**Y**	**Y**	**Y**	**7**	**77.8**	High	Included
Obi et al., 2004	480	**Y**	**Y**	**Y**	**Y**	**Y**	**Y**	**Y**	**Y**	**Y**	**9**	**100**	High	Included
Abdissa et al., 2017	2482	**Y**	**Y**	**Y**	**Y**	**Y**	**Y**	**Y**	**Y**	**Y**	**9**	**100**	High	Included
Abey et al., 2024	384	**Y**	**Y**	**N**	**Y**	**N**	**Y**	**Y**	**Y**	**Y**	**7**	**77.8**	High	Included
Abunna et al., 2023	352	**Y**	**Y**	**Y**	**Y**	**Y**	**Y**	**Y**	**Y**	**Y**	**9**	**100**	High	Included
Assefa et al., 2019	410	**Y**	**Y**	**Y**	**Y**	**Y**	**Y**	**Y**	**Y**	**Y**	**9**	**100**	High	Included
Atnafie et al., 2017	630	**Y**	**Y**	**Y**	**Y**	**Y**	**Y**	**Y**	**Y**	**Y**	**9**	**100**	High	Included
Beyi et al., 2017	525	**Y**	**Y**	**Y**	**Y**	**Y**	**Y**	**Y**	**Y**	**Y**	**9**	**100**	High	Included
Dejene et al., 2022	450	**Y**	**Y**	**Y**	**Y**	**Y**	**Y**	**Y**	**Y**	**Y**	**9**	**100**	High	Included
Fikadu et al., 2023	516	**Y**	**Y**	**Y**	**Y**	**Y**	**Y**	**Y**	**Y**	**Y**	**9**	**100**	High	Included
Mengistu et al., 2017	370	**Y**	**Y**	**Y**	**Y**	**Y**	**Y**	**Y**	**Y**	**Y**	**9**	**100**	High	Included
Sebsibe et al., 2020	502	**Y**	**Y**	**Y**	**Y**	**Y**	**Y**	**Y**	**Y**	**Y**	**9**	**100**	High	Included
Tarekegn et al., 2023	248	**Y**	**Y**	**Y**	**Y**	**Y**	**Y**	**Y**	**Y**	**Y**	**9**	**100**	High	Included
Edget et al., 2017	370	**Y**	**Y**	**Y**	**Y**	**Y**	**Y**	**Y**	**Y**	**Y**	**9**	**100**	High	Included
Mesele et al., 2023	408	**Y**	**Y**	**Y**	**Y**	**Y**	**Y**	**Y**	**Y**	**Y**	**9**	**100**	High	Included
Gutema et al., 2023	793	**Y**	**Y**	**Y**	**Y**	**Y**	**Y**	**Y**	**Y**	**Y**	**9**	**100**	High	Included
Gutema et al., 2021	583	**Y**	**Y**	**Y**	**Y**	**Y**	**Y**	**Y**	**Y**	**Y**	**9**	**100**	High	Included
Gemeda et al., 2023	539	**Y**	**Y**	**N**	**Y**	**N**	**Y**	**Y**	**Y**	**Y**	**7**	**77.8**	High	Included
Engda et al., 2023	1378	**Y**	**Y**	**Y**	**Y**	**Y**	**Y**	**Y**	**Y**	**Y**	**9**	**100**	High	Included
Parry-Hanson Kunadu et al., 2020	148	**Y**	**Y**	**N**	**Y**	**N**	**Y**	**Y**	**Y**	**Y**	**7**	**77.8**	High	Included

Donkor et al., 2008	642	**Y**	**Y**	**Y**	**Y**	**Y**	**Y**	**Y**	**Y**	**Y**	**9**	**100**	High	Included
Ka, 2014	1777	**Y**	**Y**	**Y**	**Y**	**Y**	**Y**	**Y**	**Y**	**Y**	**9**	**100**	High	Included
Bankole et al., 2014	148	**Y**	**Y**	**Y**	**Y**	**Y**	**Y**	**Y**	**Y**	**Y**	**9**	**100**	High	Included

Legend : Y= Yes; N=No; NA= Not Available

Q1. Was the sample frame appropriate to address the target population ?

Q2. Were study participants sampled in an appropriate way?

Q3. Was the sample size adequate?

Q4. Were the study subjects and the setting described in detail?

Q5. Was the data analysis conducted with sufficient coverage of the identified sample?

Q6. Were valid methods used for the identification of the condition?

Q7. Was the condition measured in a standard, reliable way for all participants?

Q8. Was there appropriate statistical analysis?

Q9. Was the response rate adequate, and if not, was the low response rate managed appropriately?
